# Attitudes of the General Public and General Practitioners in Five Countries towards Pandemic and Seasonal Influenza Vaccines during Season 2009/2010

**DOI:** 10.1371/journal.pone.0045450

**Published:** 2012-10-11

**Authors:** Patricia R. Blank, Genevieve Bonnelye, Aurore Ducastel, Thomas D. Szucs

**Affiliations:** 1 Institute of Social and Preventive Medicine, Medical Economics, University of Zurich, Zurich, Switzerland; 2 Institute of Pharmaceutical Medicine, European Center of Pharmaceutical Medicine, University of Basel, Basel, Switzerland; 3 Kantar Health, Montrouge, France; University of Pittsburgh, United States of America

## Abstract

**Background:**

Vaccination coverage rates for seasonal influenza are not meeting national and international targets. Here, we investigated whether the 2009/2010 A/H1N1 pandemic influenza affected the uptake of influenza vaccines.

**Methodology/Principal Findings:**

In December 2009/January 2010 and April 2010, 500 randomly selected members of the general public in Germany, France, the United States, China, and Mexico were surveyed by telephone about vaccination for seasonal and A/H1N1 pandemic influenza. Also, in April 2010, 100 randomly selected general practitioners were surveyed. Adult vaccine coverage in December 2009/January 2010 for A/H1N1 pandemic and seasonal influenza were, respectively, 12% and 29% in France, 11% and 25% in Germany, 41% and 46% in the US, 13% and 30% in Mexico, and 12% and 10% in China. Adult uptake rates in April 2010 were higher in Mexico but similar or slightly lower in the other countries. Coverage rates in children were higher than in adults in the US, Mexico, and China but mostly lower in Germany and France. Germans and French viewed the threat of A/H1N1 pandemic influenza as low to moderate, whereas Mexicans, Americans, and Chinese viewed it as moderate to serious, opinions generally mirrored by general practitioners. The recommendation of a general practitioner was a common reason for receiving the pandemic vaccine, while not feeling at risk and concerns with vaccine safety and efficacy were common reasons for not being vaccinated. Inclusion of the A/H1N1 pandemic strain increased willingness to be vaccinated for seasonal influenza in the United States, Mexico, and China but not in Germany or France.

**Conclusions/Significance:**

The 2009/2010 A/H1N1 influenza pandemic increased vaccine uptake rates for seasonal influenza in Mexico but had little effect in other countries. Accurate communication of health information, especially by general practitioners, is needed to improve vaccine coverage rates.

## Introduction

The World Health Organization (WHO) estimates that seasonal influenza causes between 250,000 and 500,000 deaths worldwide every year and costs between 1 and 6 million US dollars per 100,000 inhabitants [1]. In 2003, the WHO recommended reaching a vaccination coverage rate (VCR) for seasonal influenza of 75% by 2010 for high-risk populations including the elderly, nursing-home residents, people with chronic medical conditions, and pregnant women [2]. This target was officially adopted in 2009 by the European Union for the 2014/2015 influenza season [3]. The WHO also recommends vaccination for seasonal health care providers, people with essential functions in society, and children from 6 months to 2 years of age, although they did not provide specific VCR targets for these groups. In 2010, the US Centers for Disease Control Prevention Advisory Committee on Immunization Practices recommended universal influenza vaccination for all persons at least 6 months of age [4]. Their objectives for 2020 are to reach 80% vaccination coverage in children, pregnant women, and non-institutionalized working-age adults without high-risk conditions and 90% in institutionalized adults, adults with high-risk conditions, the elderly, and healthcare workers [5]. However, national recommendations as well as the reimbursement system for influenza vaccines varied from country to country (Table S1).

Despite the availability of safe and effective vaccines, seasonal influenza vaccination coverage rates for at-risk patients remains suboptimal and often far below these targets [3], [6]. To reduce the toll of the disease, vaccination campaigns must both enhance protection in the most vulnerable individuals and increase acceptance in the general population. Previous surveys in Europe and the US found that low seasonal vaccination coverage rates (VCRs) are due to no or inadequate recommendation by general practitioners (GPs), poor public awareness of influenza and influenza vaccines, a lack of proactive reminder systems, and a fear of needles [7], [8], [9], [10].

The 2009/2010 A/H1N1 pandemic dramatically raised public and decision-makers' awareness of influenza. The WHO reported more than 18,000 laboratory-confirmed deaths in 125 countries and a rate of infection between 20% and 40% in some areas [11], [12]. In the current study, during the winter of 2009/2010, we examined whether the A/H1N1 influenza pandemic affected perceptions of and coverage by influenza vaccines.

## Results

### Survey response rates

In each country, 500 randomly selected individuals in the general public completed telephone surveys in December 2010/January 2011 and in April 2011. The response rates for completed December 2009/January 2010 surveys were 11% in France, 7% in Germany, 33% in the US, 8% in Mexico, and 7% in China. For the April 2010 survey, the response rates were 5% in France, 5% in Germany, 14% in the US, 6% in Mexico, and 11% in China. Additionally, 100 GPs completed internet survey in each country during April 2011. Response rates for completed GPs survey were 63% in France, 60% in Germany, 81% in the US, 29% in Mexico, and 56% in China.

### VCRs for A/H1N1 pandemic influenza

During December 2009/January 2010, the VCR for A/H1N1 pandemic influenza among adults was close to 10% in France, Germany, Mexico, and China (Table 1). In the US, in contrast, the adult vaccination rate was substantially higher, at 41%. In the April 2010 survey, the adult VCRs were similar in all countries except for Mexico, where it increased from 13% in December 2009/January 2010 to 31% in April 2010.

**Table 1:** VCRs for A/H1N1 pandemic and seasonal influenza.

		VCR (%)				
Influenza/Age Group	Period	France	Germany	US	Mexico	China
A/H1N1 pandemic						
All adults	Dec 2009/Jan 2010	12	11	41	13	12
	April 2010	11	10	39	31	10
Children	Dec 2009/Jan 2010	17	4	46	24	37
Adults 18–64 y	Dec 2009/Jan 2010	12	11	41	13	12
Elderly adults	Dec 2009/Jan 2010	12	14	47	28	8
At-risk adults	Dec 2009/Jan 2010	17	17	37	13	14
Seasonal						
All adults	2007/08	26	25	46	22	11
	2008/09	27	26	55	33	11
	Dec 2009/Jan 2010	29	25	46	30	10
	April 2010	27	27	49	44	8
Children	2007/08	6	10	32	34	26
	2008/09	5	13	50	46	30
	Dec 2009/Jan 2010	9	14	59	54	41
	April 2010	7	12	53	62	33
Adults 18–64 y	Dec 2009/Jan 2010	29	25	46	30	10
Elderly adults	Dec 2009/Jan 2010	54	50	62	50	16
At-risk adults	Dec 2009/Jan 2010	59	43	52	44	17

In the US, VCRs were relatively constant across age groups and were the highest for all countries, at more than 40%. VCRs were also relatively constant across age groups in France, although they were all below 20%. In Germany, VCRs were also below 20% for all age groups. In this case, they were the highest among adults 18 to 64 years of age and elderly adults but very low among children (4%). VCRs in Mexico were the highest in elderly adults and children and were between 13% and 28%. In China, VCRs were highest among children (37%) but were very low in elderly adults (8%).

For high-risk adults, approximately 2 in 5 in the US were vaccinated for A/H1N1 pandemic influenza. In the other countries, less than 1 in 5 high-risk adults were vaccinated for A/H1N1 pandemic influenza.

### VCRs for seasonal influenza

For seasonal influenza, the VCRs for adults in December 2009/January 2010 ranged from 10% (China) to 46% (US) (Table 1). In the April 2010 survey, the overall adult VCR was similar for all countries except Mexico, where the rate increased from 30% in Dec 2009/Jan 2010 to 46% in Apr 2010.

VCRs by age group in each country were higher for seasonal influenza than for A/H1N1 pandemic influenza, except in China, where the overall adult VCR for seasonal influenza remained close to 10% (Table 1 ), and among French children, where it was 9% (vs. 17% for A/H1N1 pandemic influenza). In France, Germany, and the US, the VCR for seasonal influenza was highest among elderly adults, whereas in Mexico and China, the VCR was highest among children. In the US, VCRs were relatively constant across age groups, and in all cases, they were the highest of all five countries examined in this survey.

At least 2 in 5 high-risk adults were vaccinated for seasonal influenza in France, Germany, the US, and Mexico. These VCRs were higher than for A/H1N1 pandemic influenza. In China, approximately 1 in 5 high-risk adults received the vaccination, similar to the rate for A/H1N1 influenza.

Between the 2007/08 and 2009/2010 influenza seasons, the VCRs for seasonal influenza among adults remained relatively constant in each county. Although being overall steady, the VCRs were consistently highest in the US (46% in 2007/08 and 2009/2010, with a peak of 55% in 2008/09) and lowest in China (ranging from 11% to 10%) (Table 1). In contrast, VCRs among children increased over time in all countries. The highest VCRs for children were consistently in the US and Mexico and the lowest in France and Germany.

### Opinions of the general public about the 2009/2010 A/H1N1 pandemic influenza and drivers for and against vaccination

In France, Germany, and Mexico, the most common reason to be vaccinated for A/H1N1 pandemic influenza was a physician's advice or recommendation (Table 2). In the US, media advertising was the most important motivating factor, although a physician's advice was nearly as important. Motivations in China were distinct from other countries, with the most important reason for vaccination being instructions by government or health authorities, followed by media advertising.

**Table 2:** General public opinions of A/H1N1 pandemic influenza and vaccination for it.

	Number of Respondents (%)				
	France	Germany	US	Mexico	China
What was the origin of your decision to get vaccinated for A/H1N1 flu?	N = 57	N = 54	N = 228	N = 184	N = 94
Physician's advice or recommendation	23 (40)	23 (42)	84 (37)	61 (33)	9 (10)
Media	10 (18)	11 (21)	91 (40)	42 (23)	24 (25)
Government/health authorities	9 (15)	1 (2)	48 (21)	29 (16)	54 (57)
Family, friends, or relatives	3 (6)	6 (12)	57 (25)	26 (14)	16 (17)
School work/university organized vaccination	2 (3)	6 (11)	7 (3)	7 (4)	20 (21)
Pharmacist's advice or recommendation	0 (0)	1 (2)	14 (6)	6 (3)	1 (1)
Own decision/personal choice	1 (2)	3 (6)	2 (1)	8 (1)	0 (0)
Other^a^	17 (29)	12 (22)	41 (18)	28 (15)	2 (2)
How much of a threat do you consider the severity of the A/H1N1 flu to yourself?^b^	N = 500	N = 500	N = 500	N = 495	N = 500
Mild (score = 0 to 2), n (%)	155 (31)	160 (32)	90 (18)	30 (6)	85 (17)
Moderate (score = 3 to 7), n (%)	300 (60)	315 (63)	315 (63)	213 (43)	285 (57)
Serious (score = 8 to 10), n (%)	45 (9)	25 (5)	95 (19)	252 (51)	130 (26)
Mean score	4.0	3.5	5.0	6.9	5.2
How much of a threat do you consider the risk of catching the A/H1N1 flu to yourself?^b^	N = 500	N = 500	N = 500	N = 495	N = 500
Mild (score = 0 to 2), n (%)	160 (32)	195 (39)	95 (19)	45 (9)	120 (24)
Moderate (score = 3 to 7), n (%)	290 (58)	285 (57)	310 (62)	277 (56)	305 (61)
Serious (score = 8 to 10), n (%)	50 (10)	20 (4)	95 (19)	173 (35)	75 (15)
Mean score	4.0	3.2	4.9	6.1	4.5
How much of a threat do you consider the severity of the A/H1N1 flu to your children?^b^	N = 139	N = 166	N = 175	N = 152	N = 139
Mild (score = 0 to 2), n (%)	40 (29)	48 (29)	24 (14)	11 (7)	15 (11)
Moderate (score = 3 to 7), n (%)	81 (58)	106 (64)	91 (52)	65 (43)	81 (58)
Serious (score = 8 to 10), n (%)	18 (13)	12 (7)	60 (34)	76 (50)	43 (31)
Mean score	4.2	4.0	5.8	7.0	5.9
How much of a threat do you consider the risk of catching the A/H1N1 flu to your children?^b^	N = 139	N = 166	N = 175	N = 152	N = 139
Mild (score = 0 to 2), n (%)	42 (30)	52 (31)	21 (12)	14 (9)	19 (14)
Moderate (score = 3 to 7), n (%)	74 (53)	101 (61)	105 (60)	82 (54)	81 (58)
Serious (score = 8 to 10), n (%)	24 (17)	13 (8)	49 (28)	56 (37)	39 (28)
Mean score	4.4	3.8	5.7	6.3	5.5
How do you perceive the media's coverage of the A/H1N1 flu?	N = 500	N = 500	N = 500	N = 495	N = 500
Underestimates the seriousness	10 (2)	5 (1)	50 (10)	59 (12)	75 (15)
Correctly conveys the seriousness	80 (16)	115 (23)	255 (51)	208 (42)	350 (70)
Overestimates the seriousness	410 (82)	380 (76)	195 (39)	228 (46)	75 (15)

All values are n (%).

a Most common “other” reasons were: at risk/chronic health condition (all countries), for protection/prevention/worried about getting sick (France, Germany, US, and Mexico), and free/available (China).

b Responses were on a scale of 0 (no threat) to 10 (serious threat).

**Figure 1:**
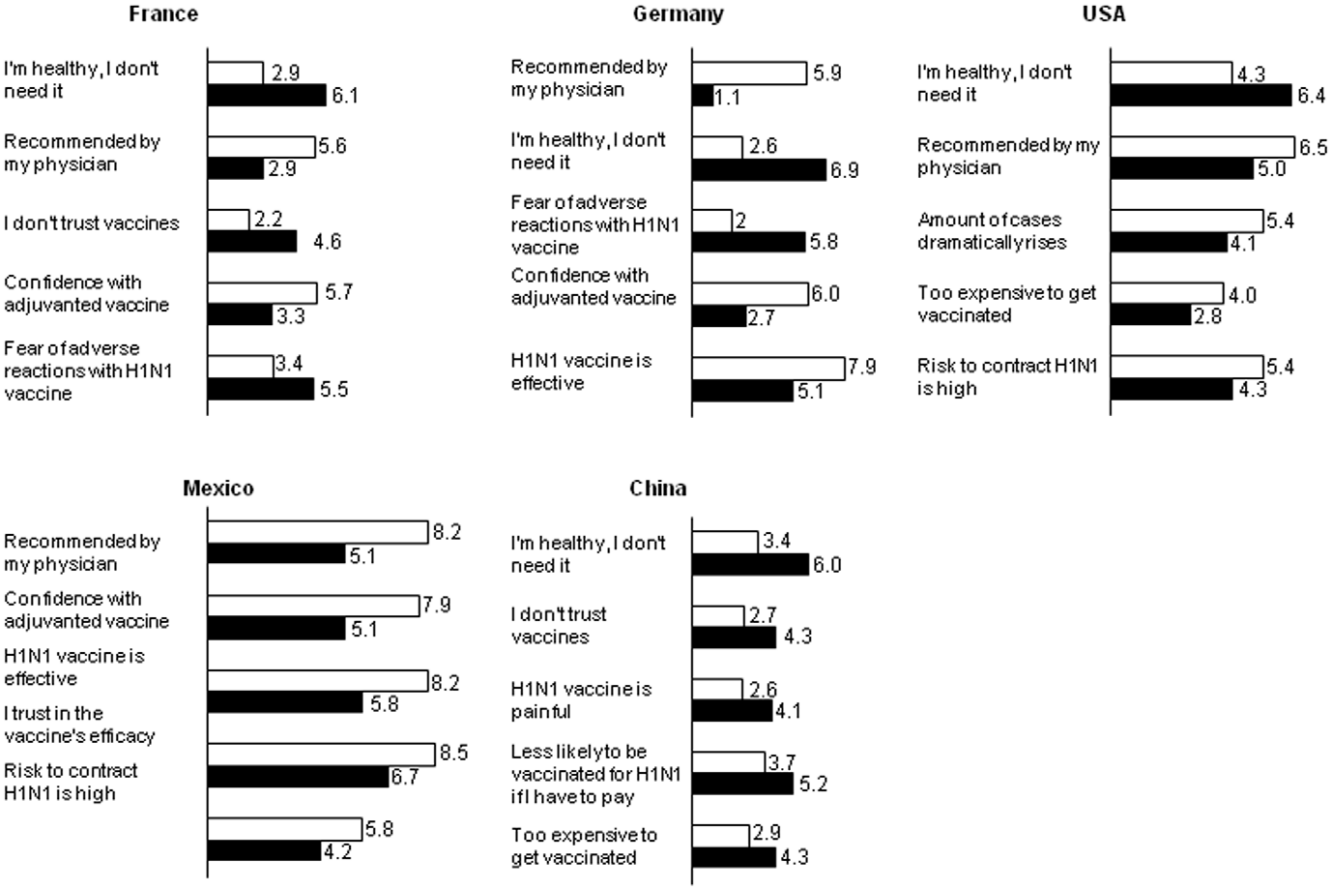
Top five difference in opinions between people vaccinated and unvaccinated for A/H1N1 pandemic influenza. General public respondents (n = 500 per country) were asked to score between 0 (totally disagree) to 10 (totally agree) with the following statements: “In most cases, A/H1N1 flu is a mild disease”, “The A/H1N1 flu vaccine works well/is efficacious”, “A/H1N1 flu vaccine is not necessary since there are effective medications to treat the A/H1N1 flu”, “The A/H1N1 flu vaccination is recommended by my physician”, “I am afraid of serious adverse reactions with A/H1N1 flu vaccine”, “A/H1N1 flu can send me to hospital”, “A/H1N1 flu vaccine is painful”, “I do not trust vaccines”, “The risk that I contract A/H1N1 flu is high”, “The A/H1N1 flu virus is mutating”, “The A/H1N1 flu vaccination is recommended by the government”, “I am healthy, I don't need it”, “It takes too much time/too complicated to get vaccinated”, “It is too expensive to get vaccinated”, “The number of A/H1N1 flu cases continues to rise dramatically”, “A/H1N1 flu complications are more severe in children than for adults”, “I would be much less likely to obtain a A/H1N1 flu vaccine if I had to pay out of pocket for the vaccine (no insurance/insurance does not cover)”, “I am confident with adjuvanted vaccine”, “I trust in the efficacy of vaccines”. Shown are the mean scores for the top five differences between vaccinated and unvaccinated individuals.

In France, the US, and China, the largest difference of opinion between those vaccinated and not vaccinated for A/H1N1 pandemic influenza was that the latter felt they were healthy and therefore did not need the vaccination (Figure 1). In Germany and Mexico, the major difference lies on the fact that several patients followed physicians' recommendation. This trend was also observed in the US and in France, but appeared as the second major difference. On the other side, key factors motivating respondents to not be vaccinated included the followings: lack of trust in vaccines (France, China), fear of adverse reactions or pain (France, China), and concerns with cost (US, China). Additionally, confidence in the vaccine's efficacy (France, Germany, Mexico) and concerns of contracting A/H1N1 influenza (US, Mexico) also appeared to be of key importance for “not vaccinated” group.

General public respondents were asked to rate the threat posed by A/H1N1 and seasonal influenza on a scale from 0 (for not a threat) to 10 (for a serious threat). More than 90% of respondents in France, Germany considered the threat posed by the severity of A/H1N1 pandemic influenza to be mild or moderate (mean score <5 in both countries) (Table 2). Most respondents in the US (81%) and China (74%) also considered this threat to be mild to moderate (mean scores ≤4). In Mexico, in contrast, just over half (51%) of the respondents considered this threat to be severe (mean score = 6.9). Patterns were similar for the threat posed by the risk of catching A/H1N1 pandemic influenza, but mean scores were slightly lower. Results were also very similar when parents were asked about the threat posed to their children by A/H1N1 pandemic influenza, although scores were higher than among adults. In summary, respondents in France and Germany tended to consider the risks associated with A/H1N1 influenza to be moderate or low, those in the US and China considered them to be moderate, and in Mexico, they considered risks to be moderate to high. Also, the threat was perceived somewhat higher for children than for adults.

### Opinions of the general public about being vaccinated for seasonal influenza the next year

Most respondents in France (56%) and Germany (62%) specified that they would probably not or definitely not be vaccinated for seasonal influenza the following year (2010/2011 influenza season), and few (30% in France and 28% in Germany) said that they would probably or definitely be vaccinated (Table 3). In the US and Mexico, in contrast, there are more respondents stating that they would probably or definitely be vaccinated than those who declared they would definitely not or probably not do so (48% vs. 27% in the US and 67% vs. 24% in Mexico). In China, the tendency is balanced as there are similar numbers of respondents for or against upcoming vaccination (40% would probably or definitely be vaccinated vs. 37% who would probably or definitely not be vaccinated.

**Table 3:** General public opinions about seasonal influenza.

	Number of Respondents (%)				
	France	Germany	US	Mexico	China
How likely are you to get a seasonal flu vaccine in the 2010/2011 season?	N = 500	N = 500	N = 500	N = 495	N = 500
Definitely won't	210 (42)	220 (44)	75 (15)	74 (15)	100 (20)
Probably won't	70 (14)	90 (18)	60 (12)	45 (9)	85 (17)
Undecided	70 (14)	50 (10)	125 (25)	45 (9)	115 (23)
Probably will	35 (7)	35 (7)	125 (25)	153 (31)	165 (33)
Definitely will	115 (23)	105 (21)	115 (23)	178 (36)	35 (7)
How likely are you to seek a seasonal flu vaccine for your child in the 2010/2011 season?	N = 144	N = 166	N = 186	N = 158	N = 146
Definitely won't	78 (54)	75 (45)	20 (11)	11 (7)	18 (12)
Probably won't	33 (23)	35 (21)	17 (9)	9 (6)	10 (7)
Undecided	13 (9)	23 (14)	35 (19)	8 (5)	17 (12)
Probably will	10 (7)	17 (10)	45 (24)	38 (24)	57 (39)
Definitely will	10 (7)	17 (10)	69 (37)	92 (58)	44 (30)

Most respondents in the US (61%), Mexico (82%), and China (69%) were probably or definitely going to have their children vaccinated, and relatively few (20%, 13%, and 19%, respectively) were definitely not or probably not. In contrast, most respondents in France (77%) and Germany (66%) were definitely not or probably not planning to have their children vaccinated the next year, while relatively few (14% in France and 20% in Germany) were definitely or probably going to have their children vaccinated.

### GP opinions of the threat posed by A/H1N1 influenza

Like the general public, GPs were asked to rate the threat posed by A/H1N1 and seasonal influenza on a scale from 0 (for not a threat) to 10 (for a serious threat). Most GPs in France (83%) and Germany (81%) considered the severity of A/H1N1 pandemic influenza to pose a mild to moderate threat to adults (mean scores ∼5) (Table 4). In the US and China, roughly similar numbers considered the threat to be moderate or serious (mean scores ∼7). In Mexico, most of them (71%) considered the threat to be serious (mean score = 8.1). In all surveyed countries, the threat posed by the risk of catching A/H1N1 was considered moderate by most respondents (mean scores 5.4 to 6.3). Results were similar when parents were asked about the threat posed by A/H1N1 pandemic influenza to their children. In summary, GPs in France and Germany tended to consider the threat posed to both adults and children by A/H1N1 pandemic and seasonal influenza as moderate, those in the US and China tended to consider the threat as moderate to severe, while GPs in Mexico tended to be more concerned about the threat they consider as severe.

**Table 4:** GP opinions about A/H1N1 pandemic influenza.

	Number of Respondents (%)^a^				
	France	Germany	US	Mexico	China
How much of a threat do you consider the severity of the A/H1N1 flu to adults?					
Mild (score = 0 to 2), n (%)	12 (12)	18 (18)	3 (3)	1 (1)	5 (5)
Moderate (score = 3 to 7), n (%)	71 (71)	63 (63)	55 (55)	28 (28)	49 (49)
Serious (score = 8 to 10), n (%)	17 (17)	19 (19)	42 (42)	71 (71)	46 (46)
Mean score	5.2	5.1	6.9	8.1	7.0
How much of a threat do you consider the risk of catching the A/H1N1 flu to adults?					
Mild (score = 0 to 2), n (%)	1 (1)	4 (4)	2 (2)	7 (7)	10 (10)
Moderate (score = 3 to 7), n (%)	87 (87)	68 (68)	75 (75)	65 (65)	70 (70)
Serious (score = 8 to 10), n (%)	12 (12)	23 (23)	23 (23)	28 (28)	20 (20)
Mean score	5.6	6.3	6.3	6.0	5.4
How much of a threat do you consider the severity of the A/H1N1 flu to children?					
Mild (score = 0 to 2), n (%)	11 (11)	17 (17)	3 (3)	3 (3)	3 (3)
Moderate (score = 3 to 7), n (%)	74 (74)	62 (62)	46 (46)	27 (27)	42 (42)
Serious (score = 8 to 10), n (%)	15 (15)	21 (21)	51 (51)	70 (70)	55 (55)
Mean score	5.4	5.4	7.3	8.0	7.4
How much of a threat do you consider the risk of catching the A/H1N1 flu to children?					
Mild (score = 0 to 2), n (%)	13 (13)	4 (4)	2 (2)	6 (6)	5 (5)
Moderate (score = 3 to 7), n (%)	84 (84)	72 (72)	73 (73)	53 (53)	55 (55)
Serious (score = 8 to 10), n (%)	3 (3)	24 (24)	25 (25)	41 (41)	40 (40)
Mean score	4.4	5.8	6.4	6.7	6.6

Responses were on a scale of 0 (no threat) to 10 (serious threat).

a N = 100 for all countries and all questions.

### Opinions of the general public towards the media during the 2009/2010 A/H1N1 influenza pandemic

Most respondents in France (82%) and Germany (76%) considered that the media overestimated the seriousness of the A/H1N1 influenza pandemic (Table 2). In contrast, most respondents and China (70%) considered that the media correctly conveyed its seriousness. In the US, the situation appeared to be somewhat contrasted. More than half (51%) stated that the media correctly conveyed its seriousness,while nearly two in five felt that they overestimated its seriousness. This contrasted trend is also observed in Mexico, where similar numbers felt that the media correctly conveyed (42%) and overestimated its seriousness (46%).

## Discussion

This survey examined influenza vaccination behaviors during the 2009/2010 A/H1N1 pandemic influenza. The study showed that 2009/2010 A/H1N1 pandemic had little effect on the uptake of the seasonal influenza vaccine, except in Mexico where it appeared to increase vaccine uptake.

In agreement with other surveys [13], [14], [15], VCRs in French and German adults for both seasonal and pandemic A/H1N1 influenza were around 10% in 2009/2010. Uptake of the seasonal influenza vaccine changed little between 2007 and 2010. As reported elsewhere [14], [16], in France, uptake of influenza vaccines and intent to vaccinate in the future was slightly better in children than adults but still remained poor. In Germany, uptake was even lower than in France, especially in children. In both France and Germany, uptake of the seasonal influenza vaccine appears to have decreased during the course of the A/H1N1 pandemic, possibly because of a negative impact of the media. Moreover, as reported in other surveys [10], [17], a physician's advice played a major role in the decision to be vaccinated in France and Germany, although the ambivalence of French and German physicians about the vaccines might have been transmitted to their patients. Generally, French and Germans do not seem to consider the benefits of influenza vaccination worth their perceived risks.

In China, VCRs for adults were consistently the lowest at approximately 10%, and they did not increase between 2007 and 2010. Vaccine uptake was better in children, and parents seemed to becoming more concerned with the threat posed by influenza to children. Lack of accurate communication of influenza-related health information seems to be the main reason for the low uptake of influenza vaccines in China. In particular, Chinese often considered themselves not at risk to be infected with influenza, or they were concerned about potential side-effects of vaccination. Finally, the 2009/2010 A/H1N1 pandemic had little or no effect on the general Chinese public's attitude towards influenza and influenza vaccination.

VCRs in adults for seasonal influenza were substantially higher in Mexico than in Germany, France, and China. Uptake of the A/H1N1 pandemic influenza vaccine was in December/January but increased by nearly 20% by the April survey. In general, Mexicans were very concerned by the threat posed by A/H1N1 pandemic influenza themselves, and even more so, they were concerned about the threat to their children. This concern appeared to have a positive effect on vaccine uptake. Also, the media, government, and GPs in Mexico appear to have been effective at conveying the seriousness of A/H1N1 pandemic and seasonal influenza.

VCRs for adults in the US were the highest for all countries for almost all categories. VCRs did not change substantially between 2007 and 2010 or between the December/January and April surveys. Vaccination for children, however, appeared to have become more important between 2007 and 2010. As in France and Germany, GPs in the US play a major role in the decision to be vaccinated, although the media may be even more important. Despite this, about half of Americans seem to resist or not bother being vaccinated and to have a negative or ambivalent opinion about vaccination for influenza. The A/H1N1 pandemic did appear to affect attitudes towards seasonal influenza vaccine in the US.

As found in other studies [18], VCRs in this survey were generally well below the WHO and US targets. VCRs for seasonal influenza in elderly and at-risk adults, where targets are at least 75% coverage [2], were between 43% and 62% in France, Germany, the US, and Mexico and only 16% or 17% in China. The WHO does not currently have targets for other groups, although in the US, 2010 recommendations were for universal vaccination, and targets for 2020 were at least 80% coverage in all groups [5]. Therefore, in all countries included in this survey, substantial efforts are still needed to reach the WHO and US targets. As found previously [18], improved or more coherent government policies and information campaigns could have a substantial effect on VCRs for influenza. In addition to that, external factors such as vaccine availability and ease to access might have a further impact on the vaccine up-take rates of influenza vaccine.

The A/H1N1 pandemic influenza appeared to increase uptake of the vaccine seasonal influenza in Mexico during 2009/2010. It has been shown, that governmental actions (such as mandatory school closure, or contain non-essential business travel) and community mitigation efforts appealing for covering sneezes, using facemasks, or social distancing initiated by the Mexican Authorities reached the majority of the population living inside and outside of Mexico city [19]. Nevertheless, confusion about information transmitted and economic barriers has also lead to inappropriate adoption of these mitigation actions. However, the immense media reports stoking fear and uncertainty about virulence and transmissibility of the virus might be one particular reason for the enhanced vaccine uptake rate seen in this country [20], [21]. Hence, it is not surprising that Mexicans tended to consider the A/H1N1 pandemic influenza a serious threat. Accordingly, many Mexicans said that inclusion of the A/H1N1 pandemic strain in the seasonal influenza vaccine would increase their willingness to be vaccinated in 2010/2011.

In contrast, in Germany, France, the US, and China, VCRs changed little between the two surveys, although they were initially higher for children in 2009/2010 than in 2008/2009, suggesting an initial effect on the uptake of seasonal influenza vaccine that was lost as the season progressed. As found in other studies [22], [23], [24], [25], [26], previous vaccination behavior appeared to be a more important factor than the A/H1N1 influenza pandemic in determining vaccination behavior.

The results of this study should be considered in light of certain limitations. All geographical areas were covered in the US, France, and Germany, but only the three largest cities were included in China and Mexico. Therefore, the VCRs in the latter two countries may be overestimated because the populations surveyed might have had easier access to the vaccines due to higher income and more local medical centers. The study may also be limited by the fact that the survey included only 500 people and 100 GPs in each country. This could result in different results than in other larger surveys or observational databases. Although the final sample was fully representative of the respective population (in terms of age, gender and region), selection bias due to the low response rates especially in Germany, China and Mexico can not be entirely ruled out. Also, the VCRs measured here are only as accurate as the respondents' recollection and honesty, and opinions could also have been influenced by the fact that the respondents were aware that they being surveyed. Indeed, VCRs in the current survey were somewhat higher than published by the US Centers for Disease Control [6] and lower than reported in Mexico [27]. However, VCRs in France and Germany were in line with previous reports [13], [14], [15].

This study confirmed that in all countries, GPs play a critical role in transmitting information about influenza and the importance of vaccination [9], [10], [17], [28]. The public appeared to generally trust and follow the advice of their GPs, both to be or to not be vaccinated, and public opinion usually mirrored GP opinion. The study also confirmed an important role for the media in the decision to be vaccinated [29], [30], [31], with a positive role in the US, Mexico, and China and a possible negative role in Germany and France. More effective, accurate, and consistent communication of health information by GPs, the media, and health authorities is needed in all countries to improve the uptake of influenza vaccines. Finally, the study showed that the A/H1N1 influenza pandemic had little effect on the uptake of seasonal influenza vaccines.

## Materials and Methods

### Study design

This was an international survey on the perceptions of seasonal influenza and seasonal influenza vaccination by adults (≥18 years of age) in the general public and GPs. The survey was performed in two parts: a first cross-sectional survey of the general public completed between December 10, 2009 and January 23, 2010 (December 2010/January 2011 survey) and a second cross-sectional survey of the general public and GPs completed between April 5 and April 23, 2010 (April 2011 survey). In order to have a global view of the study questions, surveys were carried out in all areas of France, Germany, and the US; in Mexico City, Ecatepec, and Guadalajara, Mexico; and in Shanghai, Beijing, and Guangzho, China.

All respondents gave agreement and explicit oral consent at the beginning of interviews to be included in the survey. During the interviews, a pre-defined script with the survey's purpose was used. The respondents had to give their consent which was documented by answering to the question “do you agree with the terms of this study and proceed with the study: yes/no”. Physicians gave their consent via the answer on the agreement question asked on the survey link (“Are you willing to take part in this study and provide answers for all questions that would be asked to you?”). All respondents were clearly informed that their answers would be aggregated and the aggregated results were solely intent for all types of research purposes. Both respondents and surveyors remained anonymous.

According to the guidelines of the Esomar World research codes and the European Pharmaceutical Market Research Association (EphMRA) this type of study is considered market research with no intervention and does not require the approval of an ethics committee, as this survey is a research in people, who are deemed healthy and not in the medical system [32], [33], [34]. In addition, all fieldwork was carried out by adhering to the Safe Harbor Privacy Principles of notice, choice, onward transfer, security, data integrity, access, and enforcement [35].

### Surveys

Members of the general public in each country were contacted by telephone on weekdays between 16:00 and 21:00 and on weekends between 10:00 and 18:00. Phone numbers were selected by random digit dialing, and surveys were made using a computer-assisted telephone interviewing system [36]. The first adult (≥18 years of age) in the household who picked up the telephone call became the person eligible for the interview. For each country, telephone contacts were continued until 400 completed surveys were collected from adults 18 to 64 years of age and 100 completed surveys were collected from elderly adults (≥65 years of age).

The general public surveys collected vaccination status and opinions about the A/H1N1 pandemic and seasonal influenza (such as on the disease severity, threat or risk to catch influenza for both adults and children) and about influenza vaccination in the seasons 2009/10, as well as the vaccine status of the seasons before (2007/08 and 2008/09). Responses on opinions were on a scale of 0 (no threat) to 10 (serious threat).

General or family practitioners in each country were randomly selected at their place of work by random digit dialing to participate in a computer-assisted web interview. GPs had to be performing vaccination for seasonal influenza. They were excluded from the study if practicing for less than 3 or more than 30 years. The GP surveys collected opinions about A/H1N1 pandemic and seasonal influenza and about influenza vaccination.

For both surveys, to reduce bias, the order of possible responses to multiple-choice questions were randomized (for two possible responses) or rotated (for more than two possible responses). Questionnaires were translated into the appropriate language for each country. Questionnaires were validated in three pilot interviews carried out in the US.

### Statistical analysis and considerations

Only descriptive analysis was performed. All analysis was performed using SPSS, version 16.0 (IBM, Armonk, NY). General public survey results were weighted to correct for sex ratio, age, and region of each country when differences were >3%. Weighting was not performed for the GP survey results.

## Supporting Information

Table S1 **Recommendations and national vaccine programmes in season 2009/10.** HCW, health care worker; FDA, Food and Drug Administration; GP, general practitioners. * This rule could vary according to states (Germany's 16 states were responsible for administering the immunizations in their own jurisdiction)
(DOCX)
