# Supplementary material for: Attitudes of the General Public and General Practitioners in Five Countries towards Pandemic and Seasonal Influenza Vaccines during Season 2009/2010
Source: PLoS One. 2012 Oct 11;7(10):e45450. doi: 10.1371/journal.pone.0045450 (PMC3469560; doi:10.1371/journal.pone.0045450)
Supplement: Table S1 — Recommendations and national vaccine programmes in season 2009/10. HCW, health care worker; FDA, Food and Drug Administration; GP, general practitioners. * This rule could vary according to states (Germany's 16 states were responsible for administering the immunizations in their own jurisdiction) (DOCX) [file pone.0045450.s001.docx]

|  | **France** | **Germany** | **USA** | **Mexico** | **China** |
| --- | --- | --- | --- | --- | --- |
| Population size (2010, Worldatlas) | 65,447,374 | 81,757,600 | 309,975,000 | 108,396,211 | 1,339,190,000 |
| *Start of the H1N1 vaccine program* | October 20 (HCW)  November 12 (priority groups) | October 26^th^ | October 5^th^  (Nasal spray) October 12^th^ (influenza shots) | mid December | September 21 (students participating national day) |
| *Start of seasonal influenza vaccine program* | End of September | October | End of July  (not officially, earlier than usual) | Early November | September |
| *Vaccination priority group* | 1. HCW  2. Pregnant women (6 months and over) without adjuvant (mid-November); Care providers and people living with infants lesser than 6 months of age; Health care personnel working with infants from 6 to 23 months; People aged from 2 to 64 with risk factors  3. Children and teenager from 2 to 18 years old  4. The rest of the population | 1. People working in health and safety occupations*  2. People from 6 months and older with chronic diseases *  3. Pregnant women*  4. People living in the household of at risk patients*  5. People from 6 months to 24 years*  6. People aged 25 to 59 years*  7. People above 60 years* | 1. Pregnant women; domestic members and care providers of infants lesser than 6 months of age; people aged 6 months to 24 years; health care employees and emergency medical staff; people suffering from chronic diseases  2. The sparse initial doses of the H1N1 flu vaccine were intended to be restrictively given to sub-sets like toddlers below 4 years of age  3. The rest of the population | 1. Children; elderly; pregnant women; people suffering from chronic diseases; health care employees  2. The rest of the population | 1. Patients with high risk of complications (ie. children between 6 months and 5 years old, people aged 60 and over, people suffering from chronic diseases) and health care practitioners  2. The rest of the population |
| *Time of pandemic vaccine approval by authorities* | French health authorities (Afssaps) approved vaccine against the H1N1 influenza without adjuvant (November, 16th)  H1N1 vaccine was available in week 43, 2009 | H1N1 vaccine was available in week 44, 2009 | In September 15th FDA approved the H1N1 flu vaccine | In November 24^th^, Mexico has received the first batch of 865,000 doses of vaccine against the swine flu virus | September 2009 |
| *Place of vaccination administration* | - Vaccination centers were opened from mid-January - French GPs could vaccine their patients against H1N1 flu. | - Mostly GP’s and internists were in charge of vaccination (Dependent on states) - In all states the public health departments were also in charge. | Due to the number of States it is difficult to describe a “nation distribution”. But locally, the hospitals were given vaccines and doled it out to various clinics and practices. Later on, even pharmacies and grocery stores were giving it out. | The vaccination campaign mainly took place in vaccination centers under Health ministry's supervision. | Local health bureaus and sanitary & anti-epidemic stations are the main decision-makers. They are also in charge of the vaccination. |
| *Funding* | Pandemic vaccine was free for all | Pandemic vaccine was free for all | Pandemic vaccine was paid by the government  private providers may collect copayments and other charges from patients who are insured in order to cover the costs of administering the vaccine. | Vaccine was made available for free | Provinces have their own level of local reimbursement policies |
| *Reference* | [[1]](#_ENREF_1)[2] | [[1]](#_ENREF_1)[2] | [2,3] | [2,3] | [2,3,4] |

**References**

1. Darina O’Flanagan SC, Jolita Mereckiene (2010/11) Pandemic A(H1N1) 2009 Influenza Vaccination Survey, Influenza season 2009/2010. VENICE II Consortium, Developed by Work Package No 4.

2. WHO (2009) World Health Ordganization, Pandemic influenza vaccines: current status

Pandemic (H1N1) 2009 briefing note.

3. (2010) Internal Data, Kantar Health Montrouge, France.

4. Feng L, Mounts AW, Feng Y, Luo Y, Yang P, et al. Seasonal influenza vaccine supply and target vaccinated population in China, 2004-2009. Vaccine 28: 6778-6782.
